# Electromagnetic Interference from Swimming Pool Generator Current Causing Inappropriate ICD Discharges

**DOI:** 10.1155/2017/6714307

**Published:** 2017-08-23

**Authors:** Edward Samuel Roberto, Thein Tun Aung, Atif Hassan, Abdul Wase

**Affiliations:** ^1^Wright State University Boonshoft School of Medicine, Dayton, OH, USA; ^2^Good Samaritan Hospital, Dayton, OH, USA; ^3^University of Cincinnati Medical Center, Cincinnati, OH, USA; ^4^Dayton Heart and Vascular Hospital, Dayton, OH, USA

## Abstract

Electromagnetic interference (EMI) includes any electromagnetic field signal that can be detected by device circuitry, with potentially serious consequences: incorrect sensing, pacing, device mode switching, and defibrillation. This is a unique case of extracardiac EMI by alternating current leakage from a submerged motor used to recycle chlorinated water, resulting in false rhythm detection and inappropriate ICD discharge. A 31-year-old female with arrhythmogenic right ventricular cardiomyopathy and Medtronic dual-chamber ICD placement presented after several inappropriate ICD shocks at the public swimming pool. Patient had never received prior shocks and device was appropriate at all regular follow-ups. Intracardiac electrograms revealed unique, high-frequency signals at exactly 120 msec suggestive of EMI from a strong external source of alternating current. Electrical artifact was incorrectly sensed as a ventricular arrhythmia which resulted in discharge. ICD parameters including sensing, pacing thresholds, and impedance were all normal suggesting against device malfunction. With device failure and intracardiac sources excluded, EMI was therefore strongly suspected. Avoidance of EMI source brought complete resolution with no further inappropriate shocks. After exclusion of intracardiac interference, device malfunction, and abnormal settings, extracardiac etiologies such as EMI must be thoughtfully considered and excluded. Elimination of inappropriate shocks is to “*first, do no harm*.”

## 1. Introduction

Cardiac implantable electronic devices (CIED), which include permanent pacemakers (PPM) and implantable cardioverter defibrillators (ICD), are widely used with increasing applications. Sensing circuitry inside these devices is made to detect cardiac rhythms, but they can also sense environmental signals [1]. Electromagnetic interference (EMI) includes any electromagnetic signal that can be detected by device circuitry and can potentially cause many undesirable effects on CIEDs [2, 3]. Effects of EMI on ICD operation can be varied from no effect to detrimental- incorrect diagnostics, abnormal sensing and pacing, device mode switch, and inappropriate defibrillation [4]. We describe here a unique case of extracardiac electromagnetic field interference from generator current at a public swimming pool, which resulted in false rhythm sensing and inappropriate ICD discharge.

## 2. Case Presentation

A 31-year-old female with bicuspid aortic valve, inducible sustained monomorphic ventricular tachycardia (SMVT), and arrhythmogenic right ventricular cardiomyopathy (ARVC) status post-ICD placement presented after an inappropriate ICD shock. She had Medtronic Secura DR-D224, dual chamber ICD implanted in 2011. Since the device placement, the patient never received defibrillation or cardioversion. Her ICD functioned appropriately at regular follow-ups and remote monitoring.

On the day of admission, the patient was sitting submerged in a public swimming pool. All of a sudden, she felt an electric shock which she described as getting hit at the back of the head. She had no preceding palpitations, loss of consciousness, or chest pain. The sensation reoccurred several times within a short period. The patient realized that she was being shocked by her ICD and immediately left the pool to seek medical attention.

**Figure 1:**
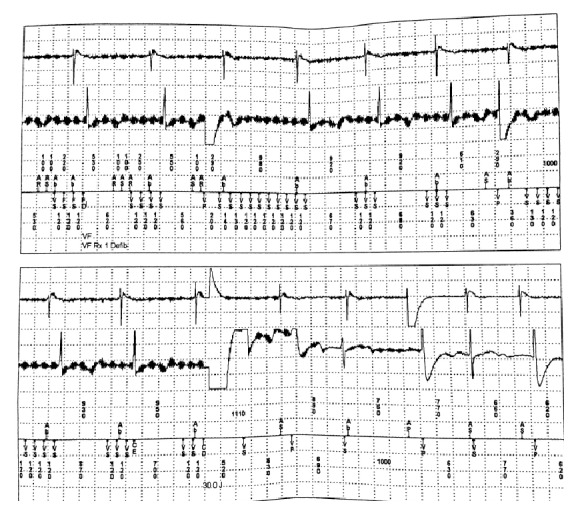
Note the high-frequency, repetitive electromagnetic (EMI) signal, exactly at 120 msec intervals. Underlying sinus rhythm and regular QRS complexes are clearly visible. The device inappropriately labeled the external AC signal as ventricular fibrillation and cardioverted with 30 J. Ab = atrial blanking period during postventricular atrial refractory period (PVARP); AP: atrial paced; AR: atrial refractory; AS: atrial sensed; CD: charge delivered; FS: (ventricular) fibrillation sensed; VP: ventricular paced; VS: ventricular sensed.

**Figure 2:**
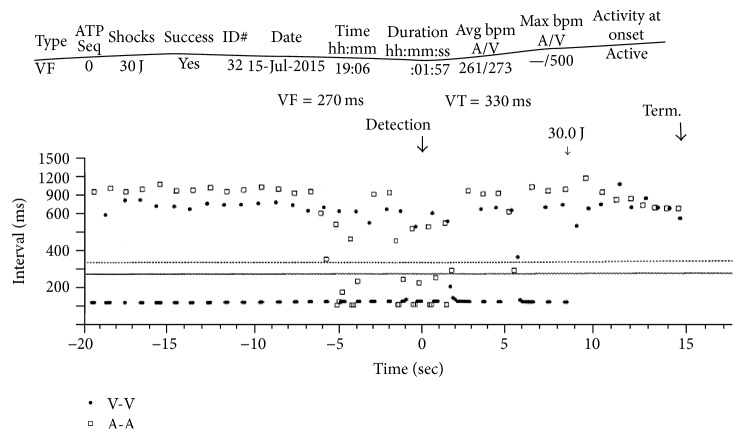
ICD histogram showing evidence of AC voltage current with a mean cycle length of 120 millisec requiring an electrical shock with resultant termination. This data lead to the diagnosis of alternating current (AC) interference by the ICD in proximity to the electrical generator, which was interpreted as a ventricular arrhythmia leading to inappropriate shock.

Intracardiac electrograms revealed a repetitive, high-frequency artifact on the ventricular channel interwoven with regular atrial and ventricular sensing (Figure 1). Electrical artifact was incorrectly sensed by the device as ventricular tachycardia/ventricular fibrillation and subsequently delivered a 30-Joule shock. Electrophysiologist reviewed the intracardiac recording and noted the signal was repetitive at exactly 120-millisecond intervals (Figure 2), raising high suspicions for alternating current from an external source. There was no evidence of device malfunction, fracture, or failure as all other device and lead parameters were within normal limits. Diagnostic information revealed ventricular sensitivity of 0.30 mV, R-waves of 4.8 mV, with ventricular lead and shock impedances of 342 and 68 Ohms, respectively. With device failure and intracardiac sources excluded, electromagnetic interference was therefore strongly suspected.

Further investigation revealed there was a submerged electric motor, which recycles the chlorinated water in and out of the swimming pool. It was subsequently discovered that there was a leak in current due to faulty grounding, which acted as the current source for electromagnetic field interference. Patient was instructed to avoid the EMI source and, thereafter, did not experience any further inappropriate ICD discharges, confirmed at repeated follow-up.

## 3. Discussion

The efficacy of ICDs in extending survival in appropriately selected patient populations has been demonstrated in primary and secondary prevention trials. Inappropriate ICD shocks due to supraventricular arrhythmia are independent predictors of mortality [5]. 829 patients were followed by Poole et al. for a median follow-up duration of 4 years. That study showed that both appropriate and inappropriate shocks predicted increased mortality [6]. Appropriate shocks were associated with hazard ratio of 5.68 and inappropriate shocks were associated with hazard ratio of 1.98. Both findings were statistically significant [7]. Data on mortality from inappropriate shocks due to EMI are lacking and inconclusive.

Whenever patients present due to ICD device firing, the device must be interrogated and the physician must review the intracardiac tracings carefully. The decision of whether it is an appropriate or inappropriate shock will have a huge influence on the prognosis and further management options. Careful thought must be given in consideration to etiologies of all kinds, both intra- and extracardiac. Inappropriate ICD shocks are due to inappropriate sensing. Abnormal sensing of supraventricular tachycardia such as atrial flutter and atrial fibrillation is the most common cause of inappropriate ICD shocks. However, electromagnetic interference and intracardiac lead dysfunction should also be considered.

Equipment malfunction encompasses lead fracture, lead displacement, lead chattering, and inappropriate settings and must be excluded before proceeding with other troubleshooting. ICD interrogation reports must be reviewed carefully. Sudden increase in lead impedance is most often due to lead fracture. Sudden drop in lead impedance is most often from insulation breakdown. After that, chest X-ray must be reviewed to exclude lead displacement or perforation. Sensing and pacing thresholds must also be checked. In our patient's case, normal pacing thresholds and lead impedances distinguished this EMI from lead integrity issues.

After exclusion of the intracardiac interference, device malfunction, and abnormal device settings, extracardiac interference such as EMI must also be excluded. Low threshold of suspicion must be present to diagnose EMI. In our patient, the intracardiac electrogram (Figure 1) clearly showed regular, repetitive artifact which is rapidly oscillated at every 120 milliseconds in both leads.

EMI is defined as oversensing of extraneous signals that adversely affect the functioning of cardiac implantable electronic devices [2, 3]. EMI can elicit undesirable responses from implanted pacemakers and defibrillators, leading to potentially devastating consequences. For example, in patients who are in complete heart block with no underlying escape rhythm, EMI can be inappropriately sensed as cardiac signals leading to asynchrony or inhibition of pacing and bradycardia or asystole. In the atria (in DDD mode) these signals can cause tracking in the ventricle leading to tachycardia. “Atrial arrhythmia” detection can lead to mode switch to VVI or DDI mode. Among patients who have ICDs, the signals can be sensed as originating in the ventricle, leading to inappropriate ICD shocks.

In a study by Occhetta et al. [7], investigation of inappropriate ICD shocks revealed seven cases due to external EMI. The sources noted in the study were an improperly grounded electric stove, electrically powered water system, hydromassage bath, electric pruner, electrocautery, and transcutaneous electric nerve stimulation. Other sources of EMI include security devices at the airport and malls, ignition systems of running motor vehicles, arc welders, electricity grids, household leakage current from improper grounding, high voltage power coils, and magnetic resonance imaging machines [8]. In our case, the alternating current leakage from the submerged electric motor in the swimming pool interacted with the ICD, resulting in an inappropriate shock.

Overall, the incidence of EMI to modern devices is low, as device technologies are constantly being developed and improved. Multiple manufacturers now have different signal detection algorithms to appropriately detect and avoid any inappropriate therapies. Current generation ICDs have built-in complex noise rejection algorithms, which reject nonphysiologic noise in the cases of lead fracture. The objective is to avoid inappropriate shocks while safely defibrillating the patients when needed. Medtronic's Lead Noise Algorithm and St. Jude Medical's SecureSense work by ensuring that ventricular sensed electrograms should be present both on RV pace-sense channel and on shock channels. If oversensing is only present on sense channel and not on shock channel, both algorithms withhold therapy. Dynamic Noise Detection is another noise rejection algorithm that is available in the newer generation Boston Scientific devices and separates the signals into low band and high band based on frequency. This potentially can prevent EMI from outside sources.

## 4. Conclusion

Several primary and secondary prevention trials have demonstrated the efficacy of ICDs in extending survival in an appropriately selected patient population. Inappropriate ICD shocks due to supraventricular arrhythmia are independent predictors of mortality. Data about survival among patients with inappropriate ICD shock due to EMI is lacking. Nevertheless, EMI remains an important potential source of inappropriate therapy with resultant psychological trauma.

For every ICD shock, the device must be interrogated. The device parameters must be reviewed carefully to make sure there is no device malfunction due to defective leads. Even after that, care must be taken to ensure that there is no abnormal sensing or inappropriate shock. Important causes of inappropriate shock include external interference such as EMI and intracardiac mechanical interference such as lead chattering. Inappropriate ICD shocks will continue unless they are recognized and solved. Inappropriate shocks inflict huge psychological trauma to the patient since the patient is shocked wide awake. Therefore, elimination of such occurrences is in keeping with our oath that is* primum non nocere* meaning “*first, do no harm.*”
